# The effect of colchicine on mortality outcome and duration of hospital stay in patients with COVID‐19: A meta‐analysis of randomized trials

**DOI:** 10.1002/iid3.562

**Published:** 2021-12-30

**Authors:** Chia Siang Kow, Learn‐Han Lee, Dinesh Sangarran Ramachandram, Syed Shahzad Hasan, Long Chiau Ming, Hui Poh Goh

**Affiliations:** ^1^ School of Postgraduate Studies International Medical University Kuala Lumpur Malaysia; ^2^ School of Pharmacy Monash University Malaysia Subang Jaya Selangor Malaysia; ^3^ Novel Bacteria and Drug Discovery Research Group (NBDD), Microbiome and Bioresource Research Strength (MBRS), Jeffrey Cheah School of Medicine and Health Sciences Monash University Malaysia Subang Jaya Selangor Malaysia; ^4^ Department of Pharmacy University of Huddersfield Huddersfield UK; ^5^ School of Biomedical Sciences and Pharmacy University of Newcastle Callaghan Australia; ^6^ PAP Rashidah Sa'adatul Bolkiah Institute of Health Sciences Universiti Brunei Darussalam Gadong Brunei Darussalam

**Keywords:** Coronavirus disease, inflammasome, NLRP3 inhibitor, SARS-CoV-2, systematic review

## Abstract

**Background:**

Overactivation of the NLR family pyrin domain containing 3 (NLRP3) inflammasome can lead to severe illness in patients with coronavirus disease‐2019 (COVID‐19). The NLRP3 inhibitor, colchicine, therefore, appears to be promising for the treatment of COVID‐19.

**Aims:**

We aimed to perform a meta‐analysis of randomized trials investigating the effect of colchicine in patients with COVID‐19.

**Materials & Methods:**

We systematically searched electronic databases and clinical trial registries (up to October 17, 2021) for eligible studies. The outcomes of interest were all‐cause mortality and duration of hospital stay. Meta‐analysis with the random‐effects model was used to estimate the pooled odds ratio (OR) of mortality and 95% confidence interval (CI). The pooled standardized mean difference of duration of hospital stay with 95% CI between colchicine users and non‐colchicine users was estimated using Cohen's d index.

**Results:**

The meta‐analyses revealed no significant difference in the odds of mortality (pooled OR = 0.76; 95% CI: 0.53–1.07), but a significant reduction in the duration of hospital stay with the use of colchicine (pooled standardized mean difference = −0.59; 95% CI: −1.06 to −0.13).

**Discussion and Conclusion:**

The ability of colchicine to reduce the length of stay in hospitalized patients with COVID‐19 is consistent with its potential to prevent clinical deterioration via inhibition of NLRP3 inflammasome. Nevertheless, such beneficial effects of colchicine did not translate into mortality benefits in patients with COVID‐19.

## Graphical abstract

Colchicine which is a potent NLRP3 inhibitor had been trialed as a potential treatment of coronavirus disease‐2019 (COVID‐19). This meta‐analysis confirms the ability of colchicine to reduce the length of hospital stay in hospitalized patients with COVID‐19, which is consistent with its potential to prevent clinical deterioration via inhibition of NLRP3 inflammasome. However, this meta‐analysis also revealed no significant difference in the odds of mortality with the use of colchicine in patients with COVID‐19.

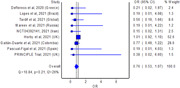

## 1 INTRODUCTION

Individuals with obesity and/or type 2 diabetes are more prone to developing severe course of illness upon acquisition of coronavirus disease‐2019 (COVID‐19).^1^ Some researchers have proposed that over‐activation of the nod‐like receptor pyrin domain containing 3 (NLRP3) inflammasome might be the underlying culprit for the development of severe course of COVID‐19 in patients with metabolic diseases.^2^ Severe acute respiratory syndrome coronavirus 2 (SARS‐CoV‐2), the causative pathogen of COVID‐19, is capable of activating the NLRP3 inflammasome, either directly or via diverse cellular/molecular signaling events. In fact, a recent study^3^ comprised of 124 patients with COVID‐19 (49% with obesity; 37% with type 2 diabetes) has confirmed the activation of NLRP3 inflammasome upon acquisition of infection with SARS‐CoV‐2 and that NLRP3 inflammasome was active in this patient population.

The NLRP3 inflammasome, a multiprotein complex in macrophages, dendritic cells, and other nonimmune cells, is a vital part of the innate immune system for antiviral host defenses. The aberrant activation of the NLRP3 inflammasome during the course of COVID‐19 leads to the production of interleukin‐1β, facilitating the development of cytokine storm and the subsequent multiorgan injury. Therefore, a well‐known NLRP3 inhibitor, colchicine, appears to be promising to be repurposed for the treatment of COVID‐19, especially in patients with concurrent obesity or diabetes. While the mechanisms of action of colchicine to inhibit NLRP3 inflammasome remain an area of ongoing research, colchicine has been previously shown to inhibit the activation of NLRP3 inflammasome.^4^ It is hypothesized that by inhibiting the activation of the NLRP3 inflammasome, colchicine reduces the release of interleukin‐1β, thus preventing the subsequent induction of interleukin‐6 and tumor necrosis factor for the recruitment of additional neutrophils and macrophages, which could otherwise cause cytokine storm.^5^

The pilot randomized controlled trial by Demidowich et al.^6^ has reported a reduction in the serum level of interleukin‐6 among patients with obesity and metabolic syndrome upon treatment with colchicine. The beneficial effect of IL‐6 inhibition in patients with COVID‐19 has been established, and the ability of colchicine to interfere also with the release of cytokines other than interleukin‐6 may be more advantageous, at least theoretically. There have been several clinical trials conducted to determine the effect of colchicine in patients with COVID‐19. We aimed to perform a meta‐analysis of randomized clinical trials investigating the effect of colchicine on the clinical outcomes in patients with COVID‐19.

## 2 METHODS

A systematic literature search with no language restriction was performed in electronic databases, including PubMed, Google Scholar, Cochrane Central Register of Controlled Trials, and preprint servers (medRxiv, Research Square, SSRN), to identify eligible studies, published up to October 17, 2021. The search strategy was built based on the following keywords and their MeSH terms: “COVID‐19,” “SARS‐CoV‐2,” and “colchicine.” The clinical trial registries of the United States (clinicaltrials.gov), China (chictr.org.cn), and the World Health Organization international (who.int/clinical-trials-registry-platform) were also searched for registered clinical trials involving colchicine in the treatment of COVID‐19, in order to identify trials with released findings. Also, the reference lists of relevant articles were reviewed to search for additional studies. Two investigators (Chia S. Kow and Syed S. Hasan) independently performed the literature screening to identify eligible studies. Studies eligible for inclusion were randomized controlled trials comparing the clinical outcomes of colchicine and its comparators in patients with COVID‐19. We excluded studies with observational design, single‐arm trials, nonrandomized trials, and trials that did not report outcomes of interest.

The outcomes of interest were all‐cause mortality and duration of hospital stay. Two investigators (Chia S. Kow and Syed S. Hasan) independently evaluated each trial, who also extracted the study characteristics. The extracted study characteristics included the first author's surname, trial design, country where the trial was performed, age of patients, the dosing regimen of colchicine, the regimen of comparator intervention, mortality events, and duration of hospital stay.

Two investigators (Chia S. Kow and Syed S. Hasan) assessed the risk of bias of the trials included with Version 2 of the Cochrane risk‐of‐bias tool for randomized trials (RoB 2),^7^ which is a standardized method for assessing potential bias in reports of randomized interventions. RoB 2 is structured into a fixed set of bias domains, focusing on different aspects of trial design, conduct, and reporting.^7^

Meta‐analysis with the random‐effects model was used to estimate the pooled odds ratio (OR) of mortality with the use of colchicine relative to non‐use of colchicine, at 95% confidence intervals (CIs). An OR of more than 1 shows increased odds of mortality while a value of less than 1 shows decreased odds of mortality, with CI inclusive of 1 indicates no significant difference in the odds of mortality. In terms of the duration of hospital stay, we estimated the pooled standardized mean difference between colchicine users and non‐colchicine users using Cohen's *d* index. When the duration of hospital stay was not reported in mean and standard deviation, we first estimated the skewness of the data distribution using median and interquartile range with the method developed by Shi et al.^8^; if the distribution of data was significantly skewed from normality, the trial was excluded for analysis of the standardized mean difference in the duration of hospital stay; if the distribution of data was not significantly skewed from normality, the mean and standard deviation of the duration of hospital stay were extrapolated from the sample size, median, and interquartile range, according to the methods developed by Luo et al.^9^ and Wan et al.,^10^ respectively. We examined the heterogeneity between studies using the *I* ^2^ statistics and the *χ* ^2^ test, and significant heterogeneity was considered at 50% and *p* < .10, respectively. Publication bias was examined using the funnel plot. All analyses were performed using Meta XL, version 5.3 (EpiGear International).

## 3 RESULTS

Our systematic literature search retrieved 689 hits, of which 450 were unique (titles retrieved after removing duplications). After screening, we selected 10 randomized controlled trials,^11^, ^12^, ^13^, ^14^, ^15^, ^16^, ^17^, ^18^, ^19^, ^20^ which included 17,976 patients with COVID‐19 (8427 patients were randomized to the colchicine group, and 9549 patients were randomized to the control group and did not receive colchicine). Nine of the included randomized trials were from Greece,^11^ Iran (*n* = 2),^12^, ^16^ Brazil,^13^ Russia,^14^ the United Kingdom (*n* = 2),^17^, ^20^ Colombia,^18^ and Spain,^19^ respectively. The remaining randomized trial^15^ was an international multicenter study performed in six countries. Details of the included studies are shown in Table 1. All of the included trials^11^, ^12^, ^13^, ^14^, ^15^, ^16^, ^17^, ^18^, ^19^, ^20^ reported mortality outcomes, whereas six trials^11^, ^12^, ^13^, ^15^, ^17^, ^19^ reported the outcome with regard to the duration of hospital stay. A differing dosing regimen of colchicine was noted across the included randomized controlled trials (Table 1); six trials^11^, ^13^, ^14^, ^15^, ^17^, ^19^ administered loading doses of colchicine for up to 5 days before the maintenance doses, whereas three trials^12^, ^18^, ^20^ administered maintenance doses of colchicine without a prior loading dose. The maximum duration of use of colchicine across the included trials^11^, ^12^, ^13^, ^15^, ^17^, ^18^, ^19^, ^20^ ranged from 6 to 28 days; the trial by Tardif et al.^14^ did not report the duration of therapy with colchicine while the trial by Mostafaie (NCT04392141^16^) did not report the dosing regimen of colchicine.

**Table 1:** Study characteristics of included trials

Study	Study design	Country	Age (median/mean)	Proportion of patients with diabetes	Body mass index (kg/m^2^; mean/median)	Regimen of colchicine in the intervention group	Regimen of comparative intervention in the controlled group	Mortality		Duration of hospital stay		Risk of bias^a^
								Colchicine users (*n*/*N*; %)	Non‐colchicine users (*n*/*N*; %)	Colchicine users (median/mean; days)	Non‐colchicine users (median/mean; days)	
Deftereos et al.^11^	Open label, randomized controlled trial	Greece	Colchicine users = 65 Non‐colchicine users = 63	N/A	Colchicine users = 27.3 Non‐colchicine users = 27.7	Colchicine + standard care Loading dose: Oral colchicine 1.5 mg followed by another 0.5 mg 60 min later if no adverse gastrointestinal effects were observed (in the case of azithromycin coadministration, a single 1.0 mg loading dose of colchicine was administered) Maintenance dose: oral colchicine 0.5 mg twice daily (reduced to once daily among patients with body weight <60 kg) until hospital discharge or a maximum of 21 days	Standard care (hydroxychloroquine/chloroquine and/or azithromycin)	1/55; 1.8	4/50; 8.0	12 (IQR: 9–22)	13 (IQR: 9–18)	Some concerns
Salehzadeh et al.^12^	Open label, randomized controlled trial	Iran	Colchicine users = 56.6 Non‐colchicine users = 55.6	11%	N/A	Oral colchicine 1 mg daily + hydroxychloroquine + azithromycin for 6 days	Placebo + hydroxychloroquine + azithromycin for 6 days	0/50; 0	0/50; 0	6.28 ± 2.51	8.12 ± 2.66	Some concerns
Lopes et al.^13^	Randomized, double‐blind, placebo‐controlled trial	Brazil	Colchicine users = 54.5 Non‐colchicine users = 55.0	39%	Colchicine users=33.5 Non‐colchicine users=29.7	Oral colchicine 0.5 mg three times daily for 5 days, followed by 0.5 mg twice daily for 5 days (if body weight ≥80 kg, the first dose was 1.0 mg; if a patient had chronic kidney disease with glomerular filtration rate under 30 ml/min/1.73 m^2^, colchicine dose was reduced to 0.25 mg three times daily for 5 days, followed by 0.25 mg twice daily for 5 days) + institutional treatment	Placebo + institutional treatment (azithromycin 500 mg once daily for up to 7 days + hydroxychloroquine 400 mg twice daily for 2 days, then 400 mg once daily for up to 8 day + unfractionated heparin 5000 UI three times daily + methylprednisolone 0.5 mg/kg/day for 5 days)	0/36; 0	2/36; 5.6	7.0 (IQR: 5.0–9.0)	9.0 (IQR: 7.0–12.0)	Some concerns
Tardif et al.^14^	Randomized, double‐blind, placebo‐controlled trial	Global	Colchicine users = 54.4 Non‐colchicine users = 54.9	20%	Colchicine users = 30.0 Non‐colchicine users = 30.0	Oral colchicine 0.5 mg twice daily for the first 3 days followed by 0.5 mg once daily for 27 days	Placebo	5/2075; 0.2	9/2084; 0.4	N/A	N/A	Some concerns
Mareev et al.^15^	Open label, randomized controlled trial	Russia	Colchicine users = 54.4 Non‐colchicine users = 54.9	12%	Colchicine users = 30.2 Non‐colchicine users = 30.6	Oral colchicine 1 mg daily for the first 3 days followed by 0.5 mg daily + antibiotic + anticoagulant; duration not reported	Antibiotic + anticoagulant	0/21; 0	2/22; 9.0	13.0 (IQR: 11.0–15.0)	17.5 (IQR: 12.5–19.8)	High
Mostafaie (NCT04392141)^16^	Randomized, placebo‐controlled trial	Iran	Colchicine users = 53.0 Non‐colchicine users = 54.1	N/A	N/A	Oral colchicine (dosing regimen not reported) and herbal phenolic monoterpene fractions + standard care	Standard care (details were not mentioned)	1/60; 1.7	6/60; 10.0	4.17 ± 1.34	6.39 ± 2.59	Some concerns
Horby et al.^18^	Open label, randomized controlled, platform trial	United Kingdom	All participants = 63.4	25%	N/A	Loading dose: Oral colchicine 1 mg followed by another 0.5 mg 12 h later Maintenance dose: 0.5 mg twice daily for 10 days in total or until discharge (once daily for patients receiving a moderate CYP3A4 inhibitor or who had renal impairment) + randomization to other treatments (convalescent plasma, monoclonal antibody, aspirin, baricitinib, or tocilizumab + usual care	Usual care (corticosteroids, azithromycin, or remdesivir)	1173/5610; 20.9	1190/5730; 20.8	10.0 (range: 5.0–28.0)	10.0 (range: 5.0–28.0)	Some concerns
Gaitán‐Duarte et al.^17^	Open label, randomized controlled trial	Colombia	Colchicine (+ rosuvastatin) users = 56.1 Colchicine (+ rosuvastatin and emtricitabine/tenofovir) users = 56.1 Non‐colchicine (emtricitabine/tenofovir) users = 54.1 Non‐colchicine (standard of care) users = 54.1	12%	N/A	Oral colchicine 0.5 mg twice daily + rosuvastatin and/or emtricitabine/tenofovir) + standard care for 14 days	Standard care (dexamethasone, ivermectin, albendazole, enoxaparin, or acetaminophen)	39/312; 12.5	50/321; 15.6	N/A	N/A	Some concerns
Pascual‐Figal et al.^19^	Open label, randomized controlled trial	Spain	Colchicine users = 51.8 Non‐colchicine users = 50.3	15%	N/A	Colchicine + standard care Loading dose: Oral colchicne 1 mg followed by another 0.5 mg 2 h later Maintenance dose: 0.5 mg every 12 h for the next 7 days and 0.5 mg every 24 h until the completion of 28 days	Standard care (dexamethasone, remdesivir, tocilizumab, baricitinib)	0/52; 0	2/51; 3.9	6.60 ± 3.86	5.76 ± 4.89	Some concerns
PRINCIPLE trial^20^	Open label, randomized controlled trial	United Kingdom	Colchicine users = 48.5 Non‐colchicine users = 61.7	11%	N/A	Oral colchicine 0.5 mg daily for 14 day + usual care	Usual care (antipyretics)	0/156; 0	9/1145; 0.8%	N/A	N/A	Some concerns

Abbreviation: IOR, interquartile range; N/A, not applicable.

^a^ Risk of bias was assessed using Version 2 of the Cochrane risk‐of‐bias tool for randomized trials.

The overall risk of bias assessed by RoB 2 is presented in Table 1. The trial by Mareev et al.^15^ had an overall high risk of bias; high risk of bias was determined for the domain of randomization because not every patient in the control group was being randomized, whereas some concerns of bias were determined for both the domain of deviations from intervention and the domain of measurement of the outcome, due to open‐label design of the trial. The remaining trials had some concerns over the overall risk of bias; the trial by Deftereos et al.,^11^ the trial by Pascual‐Figal et al.,^19^ and the PRINCIPLE trial^20^ had some concerns of bias in both the domain of deviations from intervention and the domain of measurement of the outcome, due to open‐label design of the trial; the trial by Salehzadeh et al.^12^ had some concerns of bias in the domain of randomization since the information on allocation sequence was not reported and in both the domain of deviations from intervention and the domain of measurement of the outcome, due to open‐label design of the trial; the trial by Lopes et al.^13^ had some concerns in the domain of selection of the reported results since the protocol and statistical analysis plan were not available; the trial by Tardif et al.^14^ had some concerns in the domain of randomization since the information on allocation sequence was not reported; the trial by Mostafaie (NCT04392141)^16^ had some concerns in the domain of randomization since the information on allocation sequence was not reported; the trial by Gaitán‐Duarte et al.^17^ had some concerns in the domain of measurement of the outcome since the outcome assessors were not blinded; the trial by the trial by Horby et al.^18^ had some concerns of bias in the domain of deviations from intervention due to open‐label design of the trial. All the trials above^11^, ^12^, ^13^, ^14^, ^15^ had a low risk of bias for other domains assessed.

**Figure 1:**
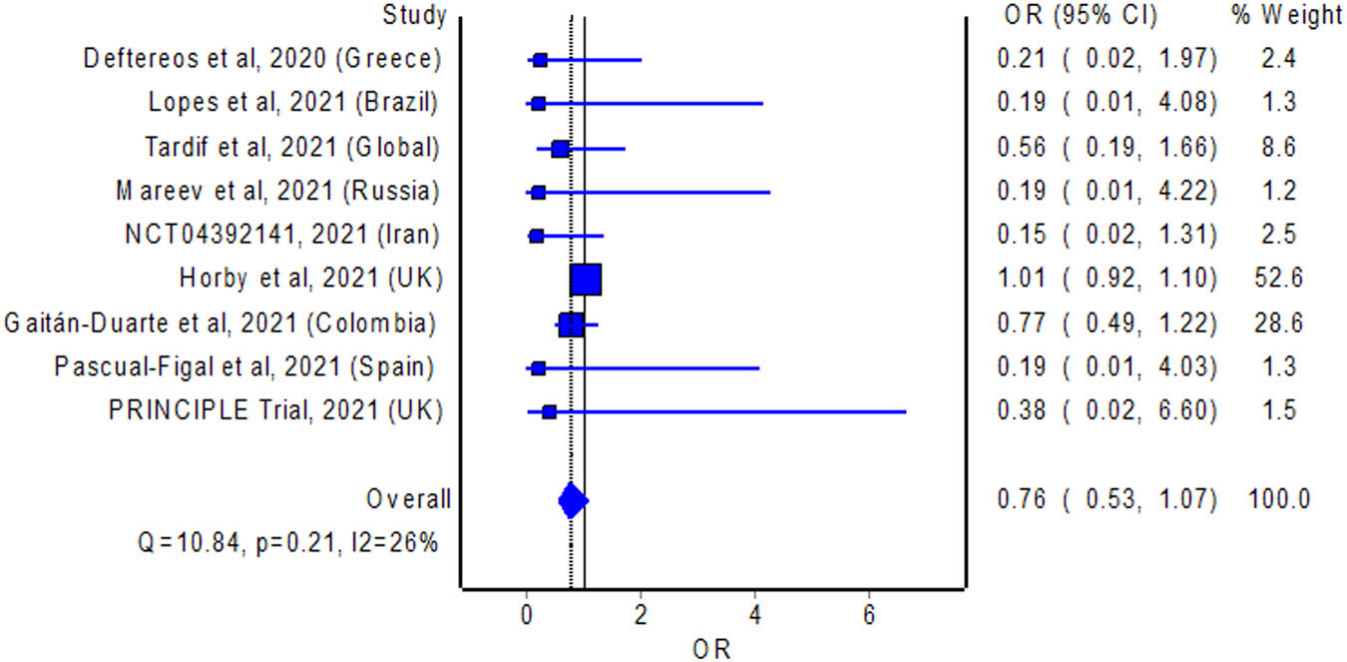
Pooled odds ratio of mortality between colchicine users and non‐colchicine users with coronavirus disease‐2019. CI, confidence interval; OR, odds ratio

**Figure 2:**
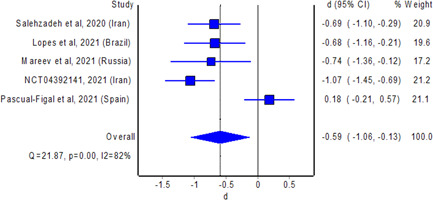
Pooled standardized mean difference of duration of hospital stay between colchicine users and non‐colchicine users with coronavirus disease‐2019. CI, confidence interval

The meta‐analysis revealed no significant difference in the odds of mortality with the use of colchicine among patients with COVID‐19 relative to non‐use of colchicine; the estimated effect though indicated mortality benefits (Figure 1; pooled OR = 0.76; 95% CI 0.53–1.07, *n* = 17,976), but is without adequate evidence against the hypothesis of “no significant difference” at the current sample size. Nevertheless, the meta‐analysis revealed a statistically significant reduction in the duration of hospital stay with the use of colchicine among hospitalized patients with COVID‐19 relative to non‐use of colchicine; the estimated effect indicated reduction of hospital stay by about 0.6 days (Figure 2; pooled standardized mean difference = −0.59; 95% CI −1.09 to −0.13, *n* = 438), with some evidence against the hypothesis of “no significant difference” at the current sample size. A funnel plot (or scatter plot) of the effect estimates from individual studies revealed no or limited bias as all studies are within a triangle centered on a fixed effect summary estimate and extending 1.96 standard errors on either side (Figure S1).

## 4 DISCUSSION

The ability of colchicine to reduce the length of stay in hospitalized patients with COVID‐19 is consistent with its potential to prevent cytokine storm via inhibition of NLRP3 inflammasome, which could prevent clinical deterioration of colchicine users with COVID‐19. Indeed, a significant reduction in the inflammatory marker, C‐reactive protein, was noticed in the trial by Mareev et al.^15^ among colchicine users (from a median of 99.4 mg/dl to a median of 4.2 mg/dl; *p* < .001), while no significant reduction was observed among non‐colchicine users. Furthermore, the trial by Deftereos et al.^11^ also reported lower maximal C‐reactive protein levels among colchicine users than non‐colchicine users (3.1 vs. 4.5 mg/dl). However, the difference is not statistically significant.

Nevertheless, such beneficial effects of colchicine did not translate into mortality benefits. It is likely that the proportion of enrolled patients with concurrent obesity and/or diabetes (ranged from 11% to 39%) who can have more pronounced activation of the NLRP3 inflammasome was too low to allow detection of mortality benefits (Table 1).^21^, ^22^ In the trial by Tardif et al.,^14^ where the participants had a median body mass index of 30.0 kg/m^2^, there was a significant reduction in the odds for a composite of death or hospitalization due to COVID‐19 in colchicine users compared to non‐colchicine users (OR = 0.75; 95% CI: 0.57–0.99). Noteworthily, the aforementioned trial^14^ investigated the use of colchicine among nonhospitalized patients with COVID‐19. Besides, the prespecified subgroup analysis reported that the odds for a composite of death or hospitalization due to COVID‐19 was trended towards a significant effect in patients with concurrent diabetes receiving colchicine (OR = 0.37; 95% CI: 0.37–1.01).^14^ Therefore, with the wisdom of hindsight, future randomized trials with colchicine should focus on the population of patients with COVID‐19 with concurrent obesity and/or diabetes, at the early (mild) stage of illness, to prevent clinical deterioration.

This study has its limitations. First, a variety of dosing regimens of colchicine was being investigated across the trials; therefore, it is not known with certainty the most appropriate regimen in patients with COVID‐19. Second, none of the included randomized trials are of high‐quality without apparent risks of bias. Third, the trials included for the estimation of pooled standardized mean difference of the duration of hospital stay were of relatively small sample size; however, there was no heterogeneity (0%) across the trials.

## CONFLICT OF INTERESTS

The authors declare that there are no conflict of interests.

## AUTHOR CONTRIBUTIONS

*Conceptualization*: Chia S. Kow and Syed S. Hasan. *Formal analysis*: Chia S. Kow, Learn‐Han Lee, Syed S. Hasan, and Long C. Ming. *Methodology*: Chia S. Kow, Dinesh S. Ramachandram, Syed S. Hasan, and Hui P. Goh. *Writing – original draft*: Chia S. Kow, Learn‐Han Lee, Dinesh S. Ramachandram, Syed S. Hasan, and Long C. Ming. *Writing – review and editing*: Chia S. Kow, Syed S. Hasan, Long C. Ming, and Hui P. Goh.

## Supporting information

Supporting information.

## Data Availability

All data generated or analyzed during this study are included in this published article.
