# Supplementary material for: The effect of colchicine on mortality outcome and duration of hospital stay in patients with COVID‐19: A meta‐analysis of randomized trials
Source: Immun Inflamm Dis. 2021 Dec 30;10(2):255–64. doi: 10.1002/iid3.562 (PMC8767506; doi:10.1002/iid3.562)
Supplement: Supplementary file 1 — Supporting information. [file IID3-10-255-s001.docx]

*Short report*

**The effect of colchicine on mortality outcome and duration of hospital stay in patients with COVID-19: a meta-analysis of randomized trials**

Running title: Meta-analysis of the effect of colchicine for COVID-19

Supplementary File S1:

**Figure S1**: Funnel plot
